# Endostatin-based anti-angiogenic therapy and immune modulation: mechanisms and synergistic potential in cancer treatment

**DOI:** 10.3389/fimmu.2025.1623859

**Published:** 2025-06-12

**Authors:** JingTao Sun, Simeng Ren, QingYun Zhao, JiaXin He, YaXuan Wang, MingHua Ren

**Affiliations:** ^1^ Department of Urology, The First Affiliated Hospital of Harbin Medical University, Harbin, China; ^2^ Department of Psychology, College of Liberal Arts, Wenzhou-Kean University, Wenzhou, China

**Keywords:** endostatin, angiogenesis, immune modulation, peptide derivatives, drug delivery

## Abstract

Cancer remains a critical global health challenge, driven by tumor angiogenesis and immune evasion. Endostatin, a collagen XVIII-derived fragment, uniquely suppresses angiogenesis and reprograms the immunosuppressive tumor microenvironment (TME), positioning it as a dual-targeting therapeutic. Despite clinical advancements with recombinant human endostatin (rhEs), challenges such as transient efficacy and delivery limitations persist. Emerging strategies integrating nanotechnology, combination therapies, and immunomodulation (e.g., TAM reprogramming, immune checkpoint synergy) aim to amplify its therapeutic potential. This review synthesizes current knowledge on endostatin’s mechanisms in angiogenesis inhibition and immune modulation. It further evaluates its clinical efficacy across solid tumors and explores innovative strategies to overcome translational barriers. By dissecting technological advancements, controversies, and synergistic opportunities with radiotherapy, chemotherapy, and immunotherapy, we aim to chart a roadmap for harnessing endostatin’s full potential in redefining precision cancer therapeutics.

## 1 Endostatin in anti-angiogenesis mechanisms therapies and beyond

### 1.1 Mechanisms of endostatin in angiogenesis inhibition

**Figure 1:**
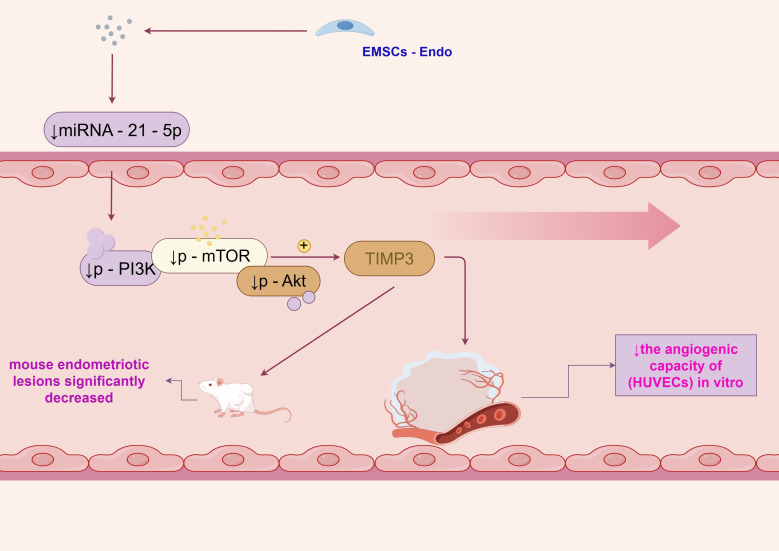
The anti-angiogenic property of endostatin.

Endostatin is a potent inhibitor of angiogenesis, and its mechanisms of action have been the subject of extensive research. One study demonstrated that endostatin - expressing endometrial mesenchymal stem cells (EMSCs - Endo) inhibited angiogenesis in endometriosis through the miRNA - 21 - 5p/TIMP3/PI3K/Akt/mTOR pathway (1). In this study, treatment with EMSCs - Endo led to a reduction in the angiogenic capacity of human umbilical vein endothelial cells (HUVECs) *in vitro*. Specifically, the miRNA - 21 - 5p level and the levels of p - PI3K, p - mTOR, and p - Akt in HUVECs and mouse endometriotic lesions significantly decreased, while TIMP3 expression significantly increased, as shown in **Figure 1**.

Beyond direct angiogenesis inhibition, endostatin’s effects extend to receptor-mediated pathways. For instance, a study explored the effect of an anti - EGFR IgG1 - Human Endostatin - P125A fusion protein on triple - negative breast cancer (TNBC) (2). The fusion protein completely inhibited the ability of HUVECs to form capillary - like structures and of TNBC cells to engage in vasculogenic mimicry and form tubes *in vitro*. *In vivo*, treatment of TNBC xenografts with the fusion protein inhibited angiogenesis and vasculogenic mimicry, reduced primary tumor growth, and decreased lung metastasis. These findings suggest that endostatin can target multiple aspects of the angiogenic process, including endothelial cell function and tumor - associated vascular mimicry.

### 1.2 Endostatin in anticancer therapy navigating challenges through genetic engineering and synergistic combinations

Endostatin, discovered over 30 years ago as a potential anti-angiogenic agent (3), has encountered obstacles in its development as an anticancer treatment. However, in 2006, a rhEs variant was approved in China for non-small cell lung cancer, marking a significant clinical milestone.

Subsequent studies have explored various strategies to improve endostatin’s properties. For example, the generation of transgenic endothelial progenitor cells (EPCs) with anti - angiogenic effects by overexpressing endostatin was investigated (4). EPCs were genetically modified to overexpress endostatin, and the results showed that these cells had an anti - angiogenic effect, as evidenced by increased endostatin expression and decreased vascular endothelial growth factor (VEGF) expression. This approach aimed to enhance the delivery and efficacy of endostatin in treating ocular neovascularization. Additionally, research on combination therapies involving endostatin has emerged, such as combining it with chemotherapy agents like cyclophosphamide in colon cancer treatment, which demonstrated an additive effect on tumor inhibition and microvessel count (5).

### 1.3 Endostatin reprograms the tumor microenvironment through vascular normalization and immune modulation in lung cancer

The tumor microenvironment (TME) plays a crucial role in tumor growth and progression, and endostatin has been shown to interact with this complex environment. In lung carcinoma models, endostatin treatment not only inhibited tumor angiogenesis but also reversed the immunosuppression of the TME (6). The microvessel density in tumors was significantly inhibited in a dose - dependent manner. Moreover, the expression levels of VEGF, interleukin (IL) - 6, and IL - 17 in tumors decreased, while interferon (IFN) - γ and hypoxia - inducible factor (HIF) - 1α expression levels increased.

In addition, the proportion of myeloid - derived suppressor cells and tumor - associated macrophages (TAMs; M2 type) decreased, while those of mature dendritic cells and TAMs (M1 type) increased, and CD8+ T cells were recruited to infiltrate the tumors. Another study investigated the effect of endostatin on the polarization of TAMs in the context of tumor vascular normalization (7). In a murine xenograft model of lung cancer, endostatin treatment led to a reduction in the number of M2 - like TAMs and Tie - 2 - expressing monocytes (TEMs) in the tumors, while the number of M1 - like TAMs showed an increase, although not statistically significant. These findings suggest that endostatin can modulate the TME by influencing immune cell populations and cytokine expression, potentially enhancing the efficacy of cancer treatments.

## 2 Clinical applications of endostatin in cancer therapy

### 2.1 Endostatin-driven combination regimens in solid tumors amplifying anti-tumor effects through radiotherapy and chemotherapy synergy

The use of endostatin in the treatment of solid tumors has been explored in numerous studies. In a study on colorectal cancer, rhEs combined with radiotherapy significantly inhibited HCT - 116 cell xenograft growth (8). The tumor growth inhibition rate in the combination therapy group was significantly higher than that in the endostatin - alone or radiotherapy - alone groups (77.67% vs 12.31% and 38.59%; n = 8 per group, P < 0.05). Immunohistochemistry results showed that treatment with endostatin or radiotherapy with endostatin induced reductions in CD31, VEGF, and HIF - 1α expression and microvascular density compared with treatment with saline.

In non - small - cell lung cancer (NSCLC), a meta - analysis of 27 randomized controlled trials (RCTs) demonstrated that the combination of rhEs, gemcitabine, and cisplatin increased the efficacy and safety of NSCLC patients (9). The effective rate (P < 0.00001) and benefit rate (P < 0.00001) of the experimental group were significantly higher than those of the control group, and the incidences of leucopenia (P = 0.79), thrombocytopenia (P = 0.39), and gastrointestinal reaction (P = 0.85) were not statistically significant. These results suggest that endostatin can be an effective adjunct in the treatment of solid tumors, especially when combined with other therapies.

### 2.2 Beyond conventional angiostatics endostatin’s unique profile and hybrid constructs in redefining anti-angiogenic strategies

When comparing endostatin with other anti-angiogenic agents, it’s important to consider its unique properties. Unlike synthetic agents, endostatin is endogenous and may have lower toxicity. A study found that combining bevacizumab and temsirolimus significantly reduced glioblastoma-induced angiogenesis at lower doses than when used alone (10).

Additionally, a recombinant CTT peptide-endostatin mimic-kringle 5 protein (AARP) demonstrated greater anti-angiogenic activity than endostatin mimic or CTT peptide-kringle 5 (RK5) *in vitro* and *in vivo* (11). AARP effectively inhibited MMP-2/9 activity, endothelial cell proliferation, migration, and tube formation, and significantly reduced tumor growth, angiogenesis, and metastasis in mouse models, while increasing survival time. These comparisons highlight the need to further explore the potential of endostatin and its combinations to optimize anti - angiogenic therapy.

### 2.3 Precision delivery of endostatin in cancers impact of combined chemotherapy and infusion strategies on survival outcomes and toxicity profiles

Clinical trials of endostatin - based therapies have provided valuable insights into its efficacy and safety. In a phase 2 single - arm multicenter open - label trial of endostatin combined with chemotherapy in small - cell lung cancer (SCLC), the median progression - free survival (PFS) was 8.0 months, the median overall survival (OS) was 13.6 months, the objective effective rate (ORR) was 61.9%, and the disease control rate (DCR) was 95.2% (12). All patients tolerated the treatment, and the main adverse reactions were myelosuppression, albuminuria, nausea, and vomiting, with the incidence of grade 3 or 4 adverse reactions being 7.2%, which could be relieved by symptomatic support treatment.

In a study comparing different administration routes of rhEs combined with concurrent chemoradiotherapy in unresectable stage III NSCLC, the continuous intravenous pumping (CIV) route showed potential advantages over the intravenous injection (IV) route (13). The median PFS, OS, local recurrence - free survival (LRFS), and distant metastasis - free survival (DMFS) in the CIV arm were 15.4 months, 38.5 months, 27.1 months, and 49.7 months, respectively, compared to 9.9 months, 24.0 months, 32.3 months, and 20.1 months in the IV arm. The incidence of hematological adverse reactions was numerically lower in the CIV arm. These results suggest that the administration route of endostatin can impact treatment outcomes.

## 3 Immune modulation by endostatin in cancer

### 3.1 Mechanisms of immune modulation by endostatin

Studies demonstrate that endostatin modulates the immune system in cancer. In lung carcinoma, endostatin reversed the immunosuppression of the tumor microenvironment (6). It decreased the proportion of myeloid - derived suppressor cells and M2 - type tumor - associated macrophages (TAMs), while increasing the proportion of mature dendritic cells and M1 - type TAMs. Additionally, CD8+ T cells were recruited to infiltrate the tumors. The expression levels of IL - 6, IL - 10, tumor growth factor - β, and IL - 17 in tumor tissue were potently decreased, while IFN - γ expression increased.

Another study investigated the effect of endostatin on the polarization of TAMs in relation to tumor vascular normalization (7). In a murine xenograft model of lung cancer, endostatin treatment led to a reduction in the number of M2 - like TAMs and TEMs in the tumors, and an increase in the number of M1 - like TAMs (although not statistically significant). These changes in immune cell populations suggest that endostatin can influence the balance between pro - and anti - tumor immune responses, potentially enhancing the body’s ability to fight cancer.

### 3.2 Endostatin as a regulator of immune checkpoints in cancer therapy

The interaction between endostatin and immune checkpoints is an area of growing interest. A study evaluated the efficacy and safety of immune checkpoint inhibitors combined with rhEs and chemotherapy as the first - line treatment of advanced non - small - cell lung cancer (14). Patients in the group receiving PD - 1 inhibitors plus rh - endostatin and chemotherapy had significantly improved progression - free survival (10.2 vs 6.5 months; p < 0.001) and objective response rate (67.2 vs 42.9%; p = 0.015), with acceptable toxicity.

This suggests that the combination of endostatin with immune checkpoint inhibitors can enhance the anti - tumor immune response. Endostatin may also influence the expression and function of immune checkpoint molecules. For example, in a study on IgA nephropathy, elevated endostatin expression was associated with disease severity, and it may be regulated by the pIgA immune complex (15). Although the direct interaction with immune checkpoints in this context was not fully elucidated, it indicates the complex relationship between endostatin and the immune regulatory network.

### 3.3 Synergistic effects of endostatin and immunotherapy

Combining endostatin with immunotherapy has shown promising synergistic effects. In a study on colorectal cancer, endostatin induced normalization of blood vessels and promoted infiltration of CD8+ T cells, which improved the efficacy of anti - PD - L1 immunotherapy (16). The antitumor effects of endostatin combined with a PD - L1 inhibitor were significantly greater than those of endostatin or a PD - L1 inhibitor alone. Endostatin treatment led to increased pseudo - diffusion parameter and microvascular volume fraction values in tumors, indicating improved intratumor microcirculation.

In addition, endostatin reversed the VEGF - mediated inhibition of the secretion of interferon (IFN) - γ from T cells. Another study demonstrated that the combination of endostatin and BRM270 not only achieved anti - tumor effects but also suppressed the proliferation of lung cancer stem cells induced by endostatin (17). These findings suggest that the combination of endostatin and immunotherapy can target different aspects of the tumor - immune interaction, leading to enhanced therapeutic efficacy.

## 4 Technological advances in endostatin delivery

### 4.1 Novel drug delivery systems for endostatin

The development of novel drug delivery systems for endostatin is crucial to improve its therapeutic efficacy. One approach is the use of gold nanoparticles (AuNPs) as a drug - delivery system for targeted tumor delivery of rh - endostatin (18). This system resulted in transient tumor vascular normalization, reduced permeability and hypoxia, strengthened blood vessel integrity, and increased blood - flow perfusion. Moreover, combination therapy with 5 - FU over this timeframe was substantially more effective than 5 - FU monotherapy.

Another study explored the use of a novel TPGS - b - (PCL - ran - PGA) nanoparticle modified with polyethyleneimine as a co - delivery system of TRAIL and endostatin for cervical cancer gene therapy (19). The nanoparticles could efficiently deliver plasmids into HeLa cells, and the cytotoxicity of the HeLa cells was significantly increased by TRAIL/endostatin - loaded nanoparticles when compared with control groups. These novel delivery systems offer the potential to enhance the targeting and efficacy of endostatin in cancer treatment.

### 4.2 Advances in endostatin formulation and bioavailability

Improving the formulation and bioavailability of endostatin is essential for its clinical success. A study optimized the high - concentration endostatin formulation by harmonizing the contributions of excipients on colloidal and conformational stabilities (20). The results showed that the soluble aggregation, fibrillation, and gelation, induced by conformational and colloidal instabilities of the protein solution, could be substantially optimized by using suitable stabilizers such as combinations of saccharides and surfactants. Avoiding the usage of salts could prevent the formation of gel and soluble aggregates at high protein concentration (e.g., 30 mg/mL) and elevated temperature (40°C).

Linking the antibody Fc domain to endostatin significantly improved endostatin’s half - life and efficacy (21). The antitumor dose of Fc - endostatin was found to be approximately 100 times less than the clinical grade endostatin, and its half - life in the circulation was weeks rather than hours, as observed for endostatin alone. These advances in formulation and bioavailability can potentially enhance the effectiveness of endostatin - based therapies.

### 4.3 Future directions in endostatin delivery technologies

Future research in endostatin delivery technologies may focus on developing more targeted and efficient systems. Transmucosal drug delivery has shown potential as an alternative route to avoid the limitations of traditional administration routes (22). Among the various transmucosal routes, the nose - to - brain delivery route has the greatest translational potential. For endostatin, exploring this route could potentially improve its delivery to specific tumor sites, especially in brain - related cancers.

The use of exosome - based drug delivery platforms targeting tetraspanins may also be a promising direction (23). Tetraspanins are involved in vesicle biogenesis and cargo trafficking, and targeting them could enhance the loading and delivery of endostatin. Additionally, advancements in mRNA vaccine delivery technologies, such as lipid nanoparticles, self - amplifying RNA, electroporation, and microneedles, could potentially be adapted for endostatin delivery, improving its efficacy and safety.

## 5 Endostatin derivatives redefining anti-angiogenic therapeutics through molecular innovation

**Figure 2:**
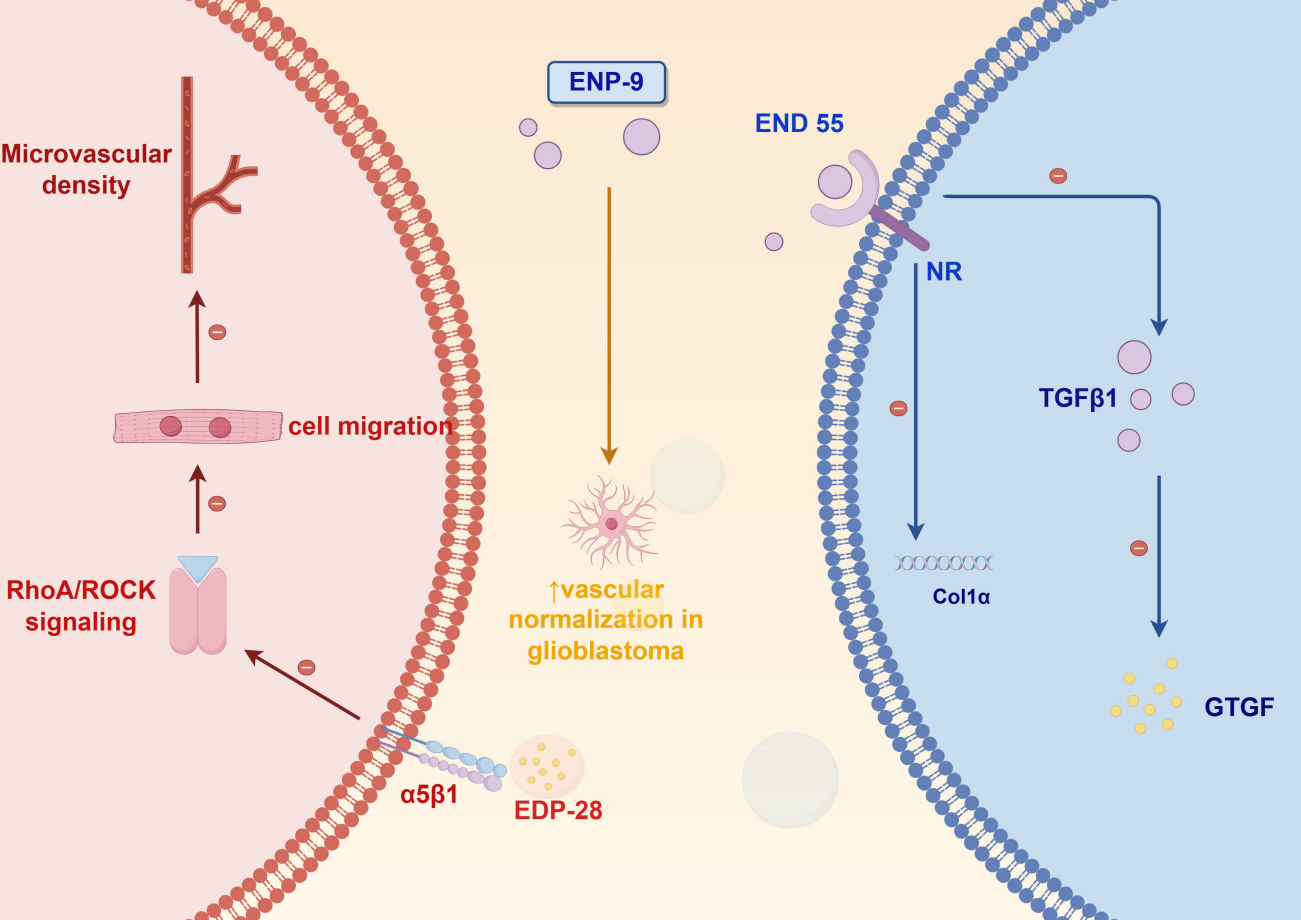
The schematic diagram of the mechanism of action of endostatin-derived peptides.

Current research advancements in endostatin-based peptide derivatives have unveiled novel insights into their angiogenesis-inhibiting properties and clinical applications for diverse pathologies, with particular emphasis on cancer therapeutics. Derived from the C-terminal domain of collagen XVIII, this endogenous inhibitor has been extensively validated for its dual capacity to impede neoplastic growth and disrupt vascular network formation. **Figure 2** highlights three distinct peptide variants developed through structural optimization, each exhibiting differentiated pharmacological profiles and molecular targets. Notably, strategic modifications of the parent endostatin molecule, including amino acid substitutions and domain truncations, have yielded compounds with enhanced target specificity and improved treatment efficacy. Mechanistic studies reveal these engineered peptides employ varied pathways ranging from endothelial cell apoptosis induction to growth factor receptor blockade, demonstrating the molecular plasticity of this therapeutic platform.

EDP-28, developed through phage display optimization, targets integrin α5β1 to inhibit endothelial cell migration by blocking signaling pathway (24). In colorectal cancer models, EDP-28 reduced microvessel densit, and its plant-based production system ensures low immunogenicity and scalable manufacturing, making it a cost-effective option for combination therapies (25, 26).

ENP-9, a site-specifically PEGylated engineered variant, demonstrates enhanced pharmacokinetic properties through extended plasma half-life. This therapeutic candidate has exhibited potent antihyperglycemic and antidiabetic efficacy while maintaining a favorable safety profile at tested dosages. These combined pharmacological characteristics support its continued investigation as a promising antidiabetic therapeutic candidate worthy of further clinical development (27).

Developed through an innovative plant-based expression platform, the therapeutic peptide END55 demonstrates significant anti-fibrotic efficacy across both murine models and human tissue systems. Mechanistic studies reveal its potent fibrosis mitigation capacity, establishing END55 as a novel therapeutic candidate for fibrotic disorders. This plant-derived production methodology not only ensures enhanced biopharmaceutical accessibility but also offers substantial cost-efficiency advantages over conventional manufacturing approaches. Importantly, END55 exhibits a favorable safety profile with no observed cytotoxic effects and lacks the drug resistance issues commonly associated with conventional chemotherapeutic agents. These combined pharmacological advantages position END55 as a breakthrough biologic with significant clinical potential for next-generation fibrosis management strategies (28). These derivatives exemplify strategies spanning targeted delivery, pharmacokinetic enhancement, and immune-angiogenic crosstalk modulation, collectively advancing precision oncology paradigms.

While these derivatives mark significant progress, challenges remain in optimizing their specificity, scalability, and integration into multimodal regimens. Future research should prioritize clinical validation of hybrid constructs and explore combinatorial approaches that exploit both angiogenesis inhibition and immune reprogramming. Additionally, leveraging advanced delivery platforms (e.g., exosomes, nanoparticles) could further enhance their precision and efficacy, bridging the gap between molecular innovation and real-world therapeutic impact.

## 6 Controversies and challenges in endostatin therapy

### 6.1 Debates on the efficacy of endostatin in different cancer types

The efficacy of endostatin in different cancer types remains a topic of debate. In non - small - cell lung cancer, some studies have shown positive results. For example, a meta - analysis of 27 RCTs demonstrated that the combination of rhEs, gemcitabine, and cisplatin increased the efficacy and safety of NSCLC patients (9). However, in other studies, the results have been less conclusive.

In a retrospective comparison of rh - endostatin plus pemetrexed/cisplatin and pemetrexed/cisplatin in advanced NSCLC, the median progression - free survival (PFS) was 10 months in the rh - endostatin group and 8.2 months in the chemotherapy group, but the difference was not statistically significant (P = 0.13) (29). In breast cancer, a prospective, randomized, phase II study showed that the combination of rh - endostatin with chemotherapy produced a higher tumor response rate without increasing toxicity in breast cancer patients, but the sample size was relatively small (30). These varying results highlight the need for further research to clarify the efficacy of endostatin in different cancer types.

### 6.2 Challenges in translating endostatin research to clinical practice

Translating endostatin research to clinical practice faces several challenges. One major issue is the production of recombinant endostatin (rEs). Its production is difficult due to its structural properties and is high - cost (31). Different expression systems, such as mammalian, baculovirus, yeast, and Escherichia coli (E. coli) expression systems, have been explored, but none of them is generally superior. Although E. coli expression system has some advantages, it still needs to overcome challenges related to protein folding and stability.

Another challenge is the short half - life of endostatin in the body. Linking the antibody Fc domain to endostatin has been shown to improve its half - life, but further optimization is needed (32, 33). Additionally, the diagnostic accuracy of endostatin for certain conditions, such as malignant pleural effusion, has been shown to be limited, with a relatively low sensitivity (34). These challenges need to be addressed to effectively translate endostatin research into clinical applications.

### 6.3 Addressing resistance to endostatin - based therapies

Resistance to endostatin - based therapies is a significant concern. A study using xenograft tumor murine models found that endostatin increased the population of ALDH+ lung cancer stem - like cells (CSLCs) by generating intratumoral hypoxia (17, 35). Endostatin also recruited TAMs, MDSCs, and Treg cells, which secrete cytokines and growth factors that increase the CSLC population. These findings imply that improving the clinical efficacy of anti - angiogenic treatments with endostatin will require the concurrent use of CSLC - targeting agents.

Another study showed that tumors can develop resistance to angiostatin gene therapy by upregulating hypoxia - inducible pathways, and blocking hypoxia - inducible factor - 1α (HIF - 1α) with antisense HIF - 1α could circumvent this resistance and augment the efficacy of anti - angiogenic therapies (36). Similar strategies may be explored to address resistance to endostatin - based therapies, such as combining endostatin with agents that target hypoxia - inducible pathways or other mechanisms of resistance.

## 7 Future perspectives in endostatin research

### 7.1 Emerging trends in endostatin and cancer treatment

One emerging trend in endostatin and cancer treatment is the combination of endostatin with immunotherapy. The gut microbiome has been shown to influence the efficacy of endostatin combined with PD - 1 blockade against colorectal cancer (37). Oral gavage of Bacteroides fragilis could salvage the antitumor effects of the combination treatment in mice with depleted microbiota, suggesting that modulating the gut microbiome may enhance the efficacy of endostatin - based immunotherapy.

Another trend is the use of endostatin in combination with other novel therapeutic approaches. For example, in non - small - cell lung cancer, the combination of recombinant endostatin with particle therapy and immunotherapy presents a promising future therapeutic triad (38). Particle therapy has different angiogenic and immunosuppressive effects compared to conventional X - ray radiotherapy, and combining it with endostatin and immunotherapy may offer enhanced treatment efficacy.

### 7.2 Potential of endostatin in combination therapies

The potential of endostatin in combination therapies is vast. In small - cell lung cancer, endostatin combined with platinum - etoposide has shown to be safe and effective, with a median progression - free survival of 8.0 months and a median overall survival of 13.6 months (12). In hepatocellular carcinoma, the combination of baculovirus - mediated endostatin and angiostatin with gemcitabine had a synergistic anti - tumor effect, reducing tumor growth, angiogenesis, and metastasis, and increasing apoptosis (39).

In advanced non - small - cell lung cancer, the combination of a PD - 1 inhibitor with rhEs not only enhanced clinical efficacy but also increased progression - free survival and the 1 - year survival rate (40). These studies suggest that endostatin can enhance the efficacy of various chemotherapy and immunotherapy agents, and further exploration of combination therapies may lead to improved cancer treatment outcomes.

### 7.3 Future research directions in endostatin and immune modulation

Future research in the domain of endostatin and immune modulation is poised to explore several critical areas. A central objective will be to achieve a more comprehensive understanding of the complex mechanisms by which endostatin exerts regulatory effects on the immune system. This entails a meticulous investigation of the interactions between endostatin and diverse immune cell populations, as well as its involvement in various cellular signaling pathways.

For example, researchers might focus on studies designed to identify the specific receptors on immune cells to which endostatin binds, along with the principal signaling molecules it activates within these cells. Such investigations would elucidate the precise mechanisms by which endostatin modulates immune functions, including cell activation, proliferation, differentiation, and cytokine secretion. Additionally, an in-depth analysis of the downstream signaling cascades triggered by endostatin would offer a comprehensive perspective on its immunoregulatory effects.

Furthermore, there exists significant potential in the development of novel combination therapies that exploit the immune-modulating properties of endostatin. These therapies could integrate endostatin with other immunomodulatory agents to augment its effects on the tumor-immune microenvironment. For instance, investigating the synergistic interactions between endostatin and agents such as immune checkpoint inhibitors or cytokines may optimize the drug combination ratios and administration schedules. This approach could enhance endostatin’s efficacy in activating anti-tumor immune responses, inhibiting tumor angiogenesis, and reconfiguring the tumor immune microenvironment, thereby potentially increasing the success rates of cancer treatments and improving patient survival outcomes.

Expanding research on endostatin to include autoimmune and chronic inflammatory diseases is essential. In autoimmune conditions, endostatin may help control immune cell activity and influence disease development and treatment. In chronic inflammation, it could regulate inflammation and angiogenesis, leading to new therapies. This broader focus could greatly increase endostatin’s potential applications and benefits for human health.

## 8 Conclusion

As a bioactive 20-kDa cleavage product of collagen XVIII, endostatin demonstrates robust anti-angiogenic activity and holds considerable promise for integration into cancer treatment regimens. Despite clinical successes, challenges such as resistance, transient efficacy, and delivery hurdles persist. Advances in genetic engineering, nanotechnology, and combination regimens—particularly with immunotherapy—hold promise for amplifying its anti-tumor effects. Future efforts should prioritize optimizing delivery systems, elucidating immune-angiogenic crosstalk, and tailoring endostatin-based therapies to individual tumor profiles. By bridging molecular innovation with clinical translation, endostatin may unlock new frontiers in precision oncology.

Through molecular redesign, endostatin-based derivatives such as EDP-28 and ENP-9 amplify their capacity to suppress angiogenesis while synergizing with immune regulatory pathways. These engineered variants enhance vascular normalization, extend pharmacokinetics, and dual-target angiogenesis-immune evasion pathways—evidenced by heightened CD8+ T cell infiltration and PD-L1 blockade efficacy. By coupling immune reprogramming with precision delivery systems, next-generation endostatin derivatives promise to transcend traditional limitations, paving the way for tailored combinatorial regimens in oncology. Future research must prioritize clinical validation of these hybrids to harness their full potential in reshaping cancer immunotherapy.
